# Successful management of rectovaginal fistula treated by endorectal advancement flap: report of two cases and literature review

**DOI:** 10.1186/s40064-015-0799-8

**Published:** 2015-01-15

**Authors:** Hirotoshi Kobayashi, Kenichi Sugihara

**Affiliations:** Center for Minimally Invasive Surgery, Tokyo Medical and Dental University, 1-5-45 Yushima, Bunkyo-ku, Tokyo 113-8519 Japan; Department of Surgical Oncology, Graduate School, Tokyo Medical and Dental University, Tokyo, Japan

**Keywords:** Rectovaginal fistula, Endorectal advancement flap

## Abstract

**Introduction:**

Rectovaginal fistula (RVF) sometimes has a difficulty in treatment. This report describes two patients who suffered from RVF.

**Case descriptions:**

One patient was a 76-year-old woman who had a RVF over 30 years after the 3rd childbirth. She underwent endorectal advancement flap (ERAF). She had a nighttime soiling after ERAF once a month, which disappeared one year after surgery. Second patient was a 23-year-old woman who had a RVF one month after the first childbirth. She underwent ERAF, and did not have any complications.

**Discussion and evaluation:**

Both patients did not develop recurrence for four years. Quality of life after ERAF was satisfactory in both patients. ERAF is a safe procedure in terms of both short and long outcomes. We also present a review of the literature concerning ERAF for RVF.

**Conclusions:**

ERAF can be a potential option as a treatment for RVF.

## Background

There is a low incidence of rectovaginal fistula (RVF) in developed countries. RVF sometimes occur after childbirth (Lowry et al. 1988; Rothenberger et al. 1982; Wise et al. 1991) and is sometimes difficult for cure. Endorectal advancement flap (ERAF) is one of options as a treatment for RVF in the world. We herein describe two patients with RVF who were successfully treated by ERAF. To our knowledge, this is the first reported cases of RVF treated by ERAF in Japan. In addition, we report a review of the literature concerning ERAF for RVF.

## Case presentation

### Patient 1

**Figure 1:**
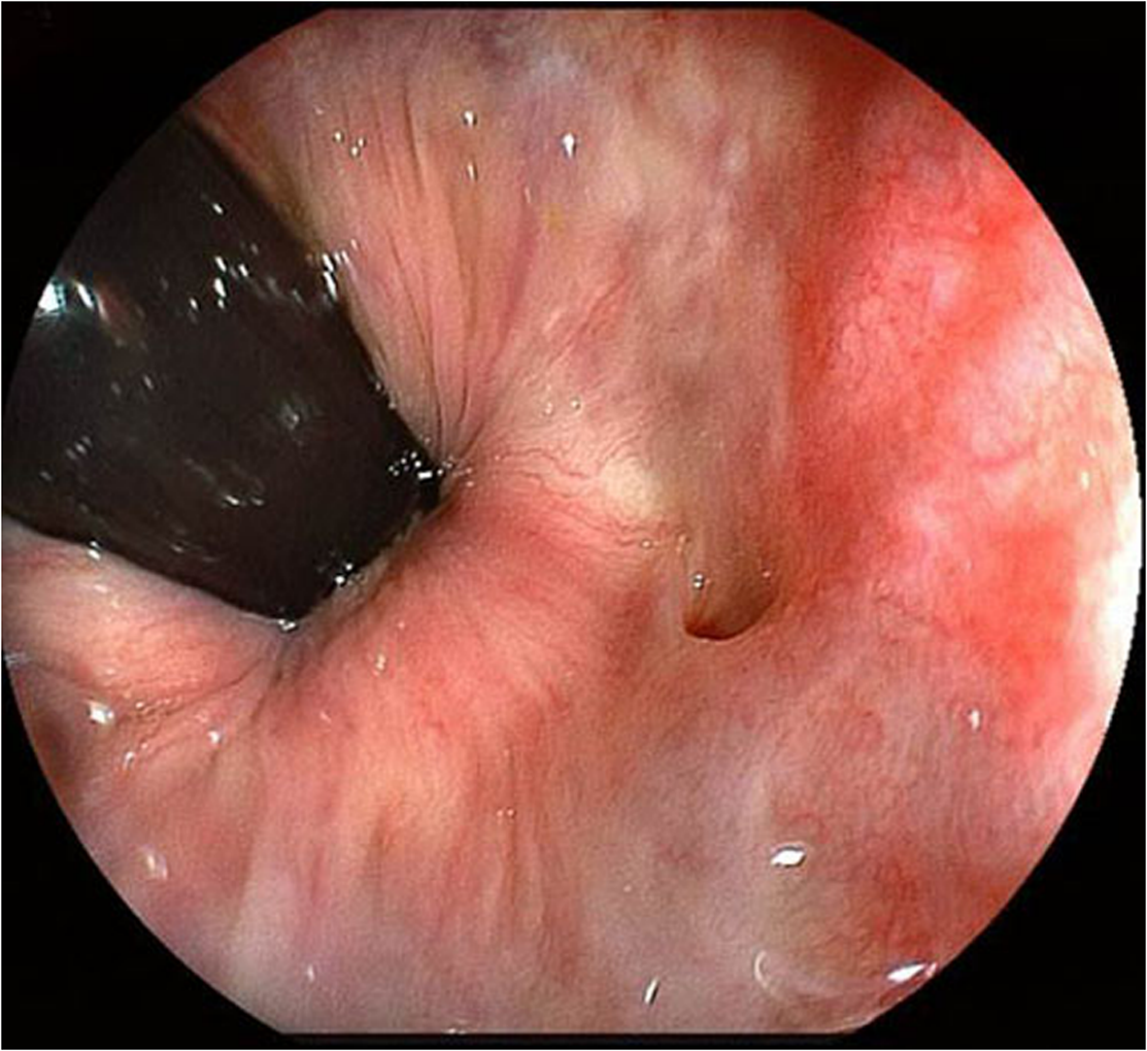
**Rectovaginal fistula seen in the colonoscopy.**

**Figure 2:**
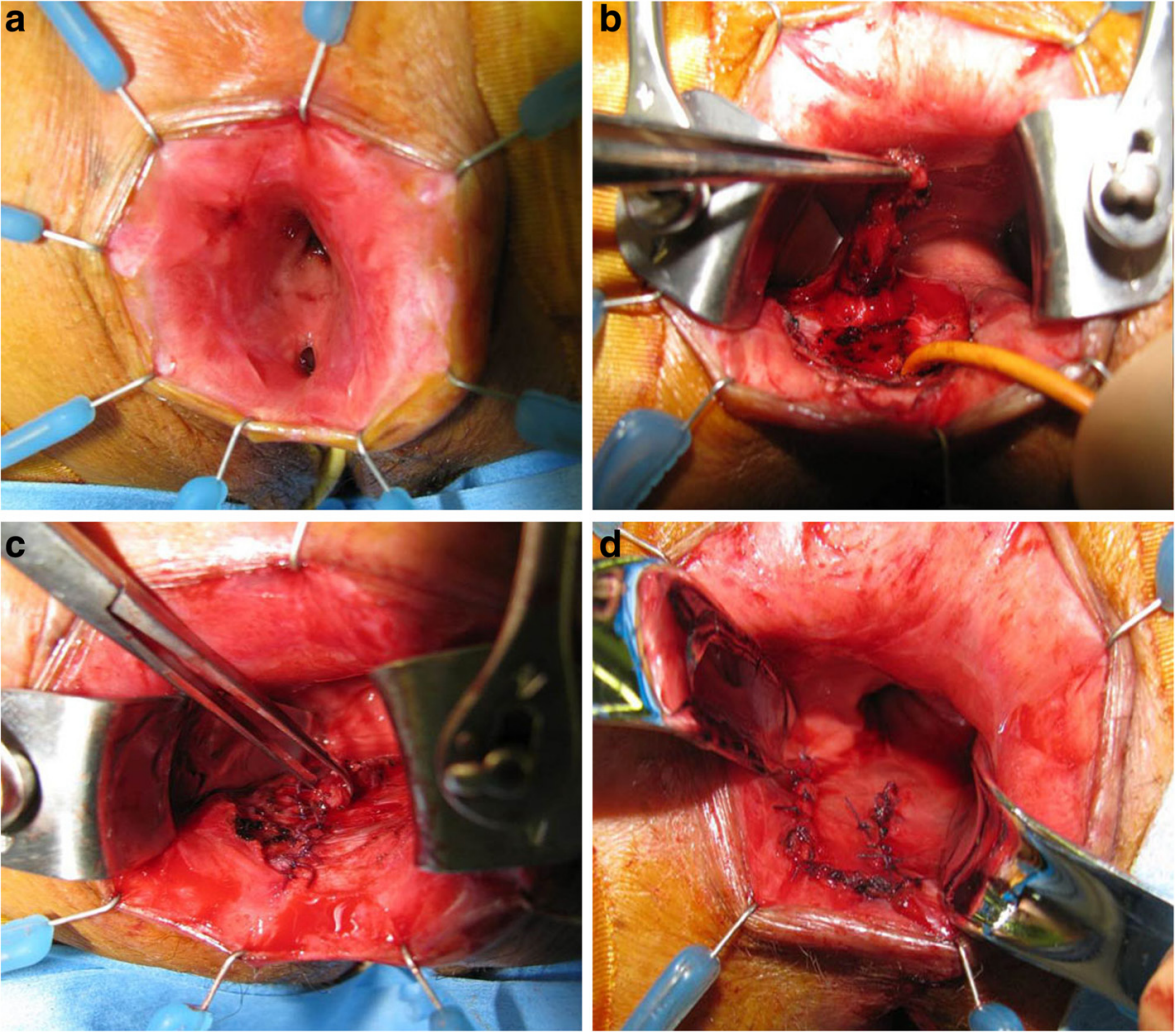
**Endorectal advancement flap for rectovaginal fistula.** Rectovaginal fistula is seen from the anus **(a)**. The flap of mucosa, submucosa, and circular muscle is raised **(b)**. Circular muscle is sutured by horizontal mattress manner **(c)**. The flap is advanced over the repaired area **(d)**. The flap is sutured in place at its apex and along its sides.

A 76-year-old woman with RVF was referred to our hospital. She suffered from RVF over 30 years after the third childbirth. A fistula was found at the level of dentate line by the anoscope examination. Similarly, colonoscopy also showed a fistula at the same level (Figure 1). She underwent ERAF under the diagnosis of RVF (Figure 2a). She was placed at the jack-knife position. The procedure of ERAF was same as previously mentioned (Rothenberger et al. 1982). In short, a four-centimeter-long flap which consisted of mucosa, submucosa, and circular muscle was outlined around the fistula (Figure 2b). The base was two times as wide as the apex of the flap for adequate blood supply. After the resection of the fistula, circular muscle was sutured by horizontal mattress manner (Figure 2c). The flap was advanced over the repaired area (Figure 2d). She had a good postoperative course and was discharged from hospital on the 7th postoperative day. She had a nighttime soiling once a month, but it disappeared one year after surgery.

### Patient 2

**Figure 3:**
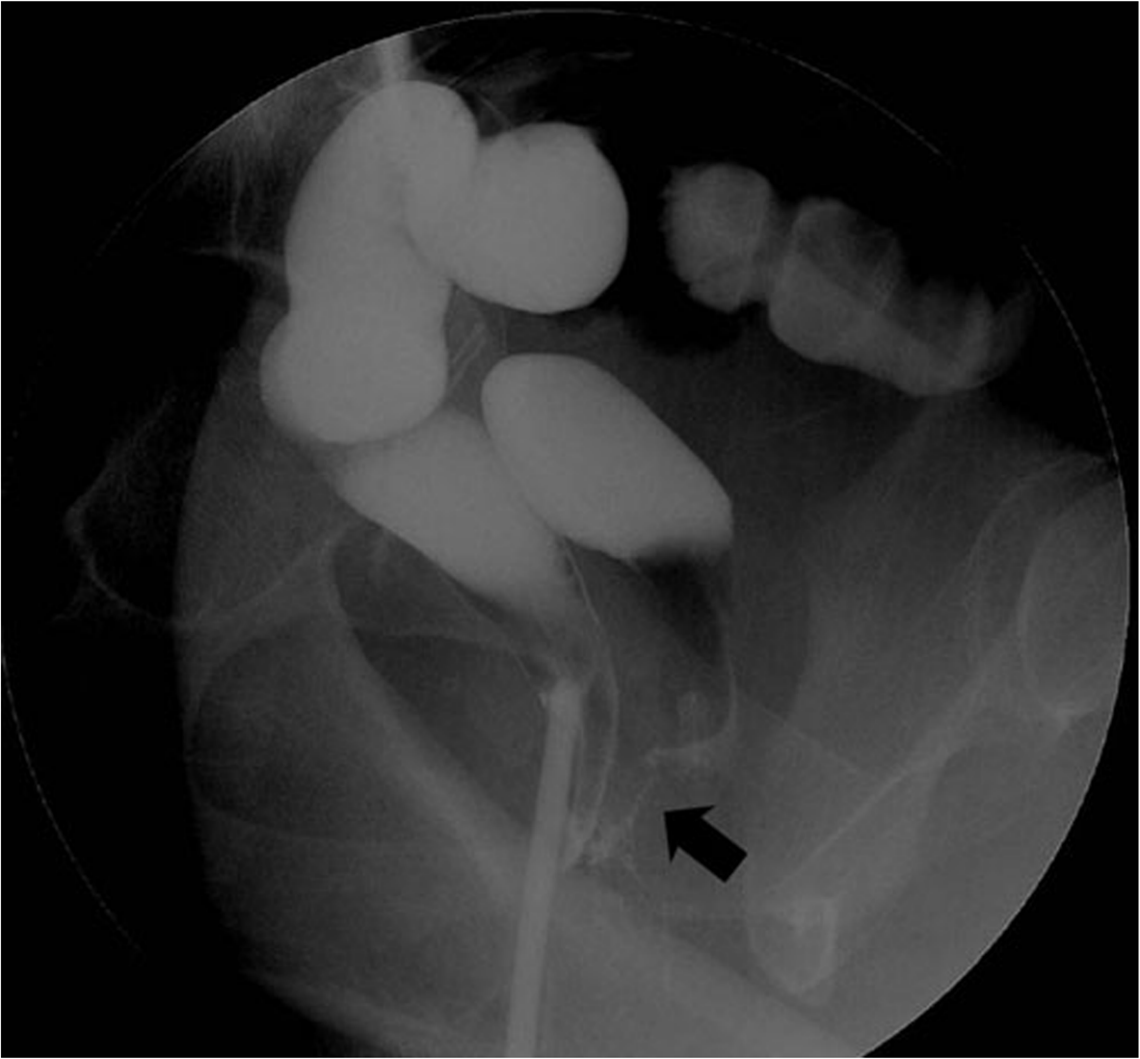
**Contrast radiography.** Black arrow shows rectovaginal fistula.

A 23-year-old woman with RVF was referred to our department during the second pregnancy. She suffered from RVF after the first childbirth. After the second childbirth via Caesarean section, contrast radiography showed the discharge from anal canal to vagina (Figure 3). She underwent ERAF and had a good postoperative course. She was discharged from hospital on the 7th postoperative day. She did not have any complications.

## Discussion

The incidence of RVF in developed counties is low, but RVF is sometimes refractory.

The incidence of RVF after vaginal delivery is 0.1% to 0.5% (Goldaber et al. 1993; Venkatesh et al. 1989). RVF at the lower level is usually caused by obstetric events. RVF at the higher level is caused by radiotherapy or rectal surgery in many cases.

Since ERAF for RVF was firstly reported in 1982 (Rothenberger et al. 1982), it has been one of the standard treatments for RVF. This procedure is a modification of mucosal flap reported by Laird (Laird 1948). Complete excision of the fistula tract is important. A flap consists of mucosa, submucosa, and circular muscle in this technique. After excision of the fistula tract, internal sphincter muscle is mobilized. Then, internal sphincter muscle is approximated in the midline without tension. In Japan, there has been no article regarding ERAF for RVF. To the best of our knowledge, this is the first report concerning ERAF for RVF in Japan. We searched the associated articles through Medline using the key words of ‘rectovaginal fistula’ and ‘endorectal advancement flap’. Thirteen English papers were matched (Table 1) (de Parades et al. 2011; Devesa et al. 2007; Joo et al. 1998; Kodner et al. 1993; Loffler et al. 2009; Lowry et al. 1988; Ozuner et al. 1996; Pinto et al. 2010; Rothenberger et al. 1982; Sonoda et al. 2002; Stern et al. 1988; Tsang et al. 1998; Wise et al. 1991). Among these, six papers included the cases of fistul-in-ano (Joo et al. 1998; Kodner et al. 1993; Loffler et al. 2009; Ozuner et al. 1996; Pinto et al. 2010; Sonoda et al. 2002).

**Table 1:** **Literature review of endorectal advancement flap for rectovaginal fistula**

**Author**	**Year**	**No. of Pts**	**Age**	**Cause**	**Size**	**Location**	**Median duration of fistula**	**Period of follow-up**	**Rate of cure**	**Complication**
Rothebnerger et al.	1982	35	35 (18-77)	Obstetric operative injury in 4 Infection in 1	Average 1 cm most were less than 2.5 cm	NA	NA	2 years	91%	NA
Lowry et al.	1988	81	34 (18-76)	An unknown cause in 6 Obstetrical injury in 74% Perineal infection in 10% Operative Trauma in7% Unknown in 8%	Less than 2.5 cm	NA	NA	NA	83%	NA
Stern et al.	1988	10	28-74	Mainly trauma	NA	NA	NA	NA	70%	NA
Wise et al.	1991	40	32.5 (20-51)	Obstetric in 25 Infectious in 8 Posttoperative in 2 Unknown in 5	NA	With in 1 cm of the dentate line	NA	NA	82.5%	Early Recurrence 2 Urinary tract infection 1 Urinary retention 1 Wound complication 3 Late Incontinence Gas/Liquid 5 Solid 2
Kodner et al.	1993	71	38 (20-71)	Obstetric injury, 48 Cryptoglandular abscess-fistula,31 Cronhn’s disease, 24 Trauma or after operation 4	NA	NA	NA	NA	84%	NA
Ozuner et al.	1996	52	38 (17-67)	Obstetric injury 13 Cryptoglandular abscess-fistula, 19 Crohn’s disease, 47 Trauma or after operation, 15 Mucosalulcerative colitis, 7	Less than 3 cm	NA	12 moths	31 months	71%	NA
Joo et al.	1998	20	40.2 (16-70)	Crohn’s disease	NA	NA	NA	17.3 months	75%	Flap retraction in 1 patient
Tsang et al.	1998	52 (62 procedures)	30.5 (18-70)	Obstetrical Obstetric injury in 5 Cryptoglandular abscess-fistula in 48	NA	NA	Na	NA	41	Bleeding in 1 patient 23%
Sonoda et al.	2002	37	42 (16-78)	Crohn’s disease in44 Trauma or after operation in 1 Other in 1	NA	NA	NA	17.1 mothd	63.6%	NA
Devesa et al.	2007	46	41	NA	NA	NA	NA	NA	100% simple fistula	NA
Loffler et al.	2009	45	NA	Cronhn’s disease Obstetric injury in 5	NA	NA	NA	48 moths	53%	NA
de Parades et al.	2011	23	45.5	Cryptoglandular disease in11 Crohn’s disease in 7 Obstetric in 18 Crohn’s in 38	NA	NA	NA	14 months	65%	NA
Pinto et al.	2010	75 procedure	41.8	Traumatic in 7 Muscosalulcerative colitis in 3 Others in 9	<0.5 cm 47.8% 0.5-1.0 cm 35.9%>1.0 cm 16.3%	Low 78.6% Middle 15.7% High 5.7%	31.2 months	20.1 months	56.3%	NA

The most frequent cause of RVF was an obstetric event (Kodner et al. 1993; Lowry et al. 1988; Rothenberger et al. 1982; Wise et al. 1991). Recently, RVF associated with Crohn’s disease has been increasing (de Parades et al. 2011; Joo et al. 1998; Kodner et al. 1993; Loffler et al. 2009; Ozuner et al. 1996; Pinto et al. 2010; Sonoda et al. 2002). The median age of RVF varied from 30.5 to 45.5 years old. Duration of disease was 12 months to 31.2 months. The average number of procedure was 1.47 (Pinto et al. 2010). Therefore, the recurrence after the treatment for RVF is problematic. The cure rate of RVF was 41% to 91% (de Parades et al. 2011; Devesa et al. 2007; Joo et al. 1998; Kodner et al. 1993; Loffler et al. 2009; Lowry et al. 1988; Ozuner et al. 1996; Pinto et al. 2010; Rothenberger et al. 1982; Sonoda et al. 2002; Stern et al. 1988; Tsang et al. 1998; Wise et al. 1991). Devesa et al. reported that the success rate of ERAF for simple rectovaginal fistula was 100% (Devesa et al. 2007). In the present study, both patients had simple RVF and succeeded in the treatment. Lowry et al. reported that the cure rates in the first, the second, and the third procedures were 88%, 85%, and 55%, respectively (Lowry et al. 1988). Another paper demonstrated that the most important factor associated with cure in patients with RVF was form: whether it was simple or complex (Devesa et al. 2007). Ozuner et al. disclosed that Crohn’s disease was associated with the highest recurrence rate among the various causes of RVF (Ozuner et al. 1996). Especially, the success rate was lower in patients with Crohn’s disease accompanying small intestinal lesion (Joo et al. 1998). Therefore, ERAF is considered useful in patients with simple RVF after obstetric event.

Accurate diagnosis is mandatory before surgery. However, contrast radiography for rectovaginal fistula is sometimes difficult, because contrast radiography can only detect high rectovaginal fistulae unless the applicator does not extend the level of the internal anal opening.

There are some limitations in this study. There are a variety of treatments for rectovaginal fistula. Transverse transperineal approach as well as transabdominal approach exists. In some cases, Martius flap can be another option for RVF (Elkins et al. 1990). However, we could not compare these techniques in this study, because of lack of RVF cases.

## Conclusions

We experienced two patients with RVF who were treated by ERAF successfully. Simple RVF after obstetric event is considered good indication for ERAF. ERAF can be a potential option for RVF in Japan.

## Consent

Written informed consent was obtained from the patient for the publication of this report and any accompanying images.
